# The Influence of Electrical Currents upon the Nervous System

**Published:** 1870-05

**Authors:** 

**Affiliations:** From Robins’ Journal de l’Anatomie et de la Physiologic normale et Pathologique; From Robins’ Journal de l’Anatomie et de la Physiologic normale et Pathologique


### Original Translations.

ARTICLE I. — *The Influence of Electric Currents upon the Nervous System.* By MM. LEGROS and ONIMUS. From Robins' Journal de l'Anatomie et de la Physiologie normale et Pathologique.

EXPERIMENTS UPON PERIPHERAL NERVES—PRELIMINARY.— In reading the majority of authors who have investigated the influence of electricity upon the nervous system, one is struck with the great number of experiments which have been made upon this subject, as contradictions which exist not only between different experimenters, but frequently with the same experimenter. Perhaps no department of science comprises so many opposing facts.

This is readily explained, for the conditions of experimentation are very complicated; it is necessary, indeed, to take into consideration not only the force of currents but even their direction, as well as the functions of the nerve, its state of excitability, and its relations with the nervous centres. By treating each case separately and by citing only the facts which experiment has placed beyond doubt, we hope to be able to reduce all these facts to a few simple laws, which may be readily retained, and which should constantly guide the physician in his therapeutic application.

### § 1.—*The Influence of Continuous Currents upon Motor Nerves.*

We lay down at first, the following principles: When mixed nerves in communication with the spinal marrow are electrized, the contractions may be reflex; it therefore becomes necessary, in order to distinguish clearly that which is due to the direct influence of the motor nerve, to separate the nerve from the cord. It is useless to say that, on the contrary, the nerve should be united to the cord, when the influence of electricity upon the sensitive nerves is to be examined.

#### A.—*Direct, Centrifugal or Descending Current.*

When upon any nerve, the sciatic for example, the electrodes of a pole are placed in such a manner that the positive pole shall be placed above the negative, a contraction will be observed at the moment when the circuit is closed, and in certain cases, at the moment when the current ceases. During the whole time that the current circulates, there is no contraction unless there should be variations in its intensity.

If a very feeble current is employed, or if the frog is already exhausted, a contraction is obtained only at the moment of closing, and none at the opening of the current.

If the current be of medium intensity, or even if the current be quite weak and the frog very active, a contraction is obtained at the closing, and at the opening of the current. The contraction produced at the opening of the current is always weaker than that which occurs at its close.

B.—*Inverse, Centripetal or Ascending Currents.*

If an inverse or ascending current be caused to act upon a nerve, contraction occurs only at the opening of the current, when the current is weak. When the current is strong, contractions occcur, as in the case of the direct current, both at its opening and at the closing.

Upon measuring with the dynamometer the contractions resulting from the application of an electric current to a nerve, it will be perceived that the contraction excited by the introduction of a direct current into a nerve, is always perceptibly stronger than that excited under similar conditions by the inverse current. (Matteucci.)

By using, at first, a current weak enough to produce no sensible excitation, then by gradually augmenting its intensity, the phenomenon of contraction is first observed when the direct current begins to pass; the inverse current, in order to produce contractions, at the moment of its cessation, must be a little stronger. (Matteucci.)

The direct current, therefore, acts more energetically upon muscular contraction than the inverse. This law explains why, in the experiment of Mariane, in passing the current from one arm to another, the contraction is most energetic in the arm in contact with the negative pole. It is, in fact, in this limb that the current is direct.

The same reason explains the majority of the facts observed by M. Chauveau.

The physiologist has attempted to establish as a law, that the electric current acts only at its point of exit, that is to say, on the side of the negative pole. In this case, again, there should be nervous filaments traversed by the direct current, which fact evidently determines more energetic contractions in the muscles situated near the negative pole.

We may conclude from all these facts that the direct or descending current is that which acts most energetically upon the motor nerve.

§ 2.—*The Influence of Continuous Currents upon Sensitive Nerves.*

Excitation of sensitive nerves can have as its consequence only the phenomena of pain or of contractions by reflex action. In every case, in order that sensitive nerves may function or determine an action resulting from their excitation, it is necessary that they should be united to the cord. They are, so to speak, in relation to the cord, what the motor nerves are in relation to the muscles; they transmit their excitation to the nervous cells of the cord in the same manner as the motor nerves transmit it to the muscles, and both call into action the elements in which they terminate, and of which they are the natural excitors.

A.—*Direct Current.*

The direct current acts very little upon sensitive nerves; in mixed nerves it excites especially, as we have just said above, contractions in the muscles which receive the branches of the motor nerve. In general, it determines very few of the phenomena of sensibility, at the moment of closing, but very often the contrary occurs at the moment of opening. We shall have to revert to this last action, when we study the currents of polarization from a physiological point of view. The inverse current determines phenomena of sensibility, and, in addition, muscular movements in the back and the upper limbs. At the moment of its application the animal frequently utters cries, and in the case of the frog, as also of the dog, and even in man, there is, in their relations to sensation and pain, a very great difference between the descending and the ascending current.

When the nerve is much exhausted, the inverse current determines but very little action in the sensitive nerve, but its excitability disappears much sooner than that of the motor nerve.

We have endeavored to isolate, completely, the influence of electricity upon the sensitive nerves of limbs. To this end one of us endeavored to utilize the properties of curare, which leaves, during a certain time at least, the sensitive nerves intact, and which does not paralyze the motor nerves of a limb, if by means of a ligature the arterial circulation, and, consequently, the passage of the poison into the tissues of the limb, be intercepted.

By interrupting the access of blood to the right leg in a frog killed by curare, we have thus, in all portions of the body, sensitive nerves capable of transmitting impressions, but there are no motor nerves intact except in the right leg. The contractions of the muscles of this leg will be therefore purely reflex, if the nerves of the opposite leg, which rests completely immobile, be excited.

By applying a descending current to the sciatic nerve of the paralyzed leg, contractions occur in the healthy leg opposite, at the closing of the current, and none at the opening. An ascending current determined contractions at the opening of the current, and none at the closing. However, during the first moments of the experiment, the ascending current determined especially contractions in the healthy leg, at the moment of closing the current; but these contractions disappeared quite rapidly. Hence, we concluded that reflex contractions were determined by electric excitation of sensitive nerves at the moment of the closing of a descending, and at the moment of the opening of an ascending current. Subsequently, repeating the same experiments, we have discovered in the operative procedure, a condition important to be noticed, and which led us into error. We took every precaution to avoid derived currents. We wiped the nerve dry, placed it upon a perfectly dry piece of glass, separated completely from each other the two sciatic nerves, and consequently believed ourselves to be under conditions the most favorable to obtain contractions purely reflex. But, in spite of all these precautions, the greater portion of the results obtained was due to derived currents, as we perceived more recently. The two legs, instead of being completely separated, were united at their upper portion by the bones of the pelvis. This sufficed to enable the current to circulate from one electrized leg to the other, and to determine derived currents, which induced contractions. By effectually cutting the pelvis, and separating the two legs, these same contractions were no longer obtained, and it was sufficient to induce their reappearance, to extend a moist thread from one to the other.

We shall have to revert to the importance of derived currents; but we shall at once, in this paragraph, establish that we have been wrong in announcing that the descending current determined in nerves purely sensitive, contractions at its closing, and that the ascending current determined them at its opening. The contrary occurs, and the only reflex contractions which we obtain in this case, are those which occurred at the moment of the closing of the ascending current.

Upon nerves of sense, electric currents determine different effects, according to the nature of the nerve; but the effects produced remain, frequently, during the whole time of the passage of the electricity. When we apply, upon the side of the head, a current of medium intensity, and in nervous persons, even a feeble current, there is experienced in the mouth, during all the time that the current circulates, a very decided metallic taste. Most persons compare this taste to that of iron, and often retain it several hours after electrization. It is difficult to explain the production of this special taste. Is it a peculiar influence upon the nerves of taste, or does it occasion in this case a slight chemical decomposition of the iron enclosed within certain elements of the organism?

Applied in the neighborhood of the acoustic nerve, voltaic currents give origin to phenomena of buzzing, especially in persons a little deaf; these buzzings are uniform during the whole time occupied by the passage of the current.

These phenomena are important, because they prove that continuous currents act upon sensitive nerves during the whole duration of their passage, and not alone at the closing and opening.

The optic nerve manifests the phenomena of phosphenes only at the moment of the application and of the cessation of the current.

The facts which we reported at the beginning of this paragraph, which are elsewhere confirmed by those which we shall examine in the following paragraph, enable us to establish the following law: *The inverse or ascending current is that which acts most energetically upon sensitive nerves.*

§ 3.—*The Influence of Continuous Currents upon Mixed Nerves.*

In a mixed nerve we should recognize the two preceding laws, for we operate, as it were, at the same time upon motor and sensitive nerves: it is thus that we obtain, at once, the contractions to which we have already referred, and phenomena of sensibility, evidences of pain, and reflex contractions in other muscles. The phenomena of sensibility take place especially at the cessation of the direct current and at the closing of the inverse current. By preparing a frog in the manner of Galvani, and by plunging each of its paws into a glass filled with ordinary water, in connection with one of the poles of the pile, the influence of the direction of the currents upon muscular contractions may be very readily studied. In this experiment, in fact, one of the paws, that which is plunged into the glass in which is the positive pole, is traversed by an ascending, the other, on the contrary, by a descending current. In this case, as was recognized long since by Aldini, Marianini, Ritter and others, in the first moments contractions occur in the two legs at the moment of the closing and of the opening of the current. The contractions at the closing are always more energetic than those which occur at the opening.

When the current is very feeble, and especially when the nerve has lost a little of its excitability, contractions occur only at the closing in the leg traversed by the direct, and the opening in the leg traversed by the inverse current.

These contractions are due to the excitations of the nerves which unite the two legs; that is to say, the electric current acts upon the nervous function, and that the nerves do not serve simply as conductors. Indeed, when the same experiment is made upon a frog poisoned with curare, or in which the nerves have been crushed, these alternations are no longer obtained, and, except by the employment of a very strong current, and by moistening the nerves, no muscular contraction is perceptible. This experiment is likewise one of the most conclusive to demonstrate that the nerve-substance is a bad conductor of electricity; for in this case, its excitability being destroyed, it should act only as a conducting medium; if the nerve be replaced by any other tissue, or by a moistened thread of silk, much more energetic muscular contractions are obtained.

By placing a frog, prepared after the manner of Galvani, astride of two glasses into which are plunged the rheaphores of a pile, besides the alternations of contraction, already indicated, another phenomenon of great value may be observed, as follows: By prolonging the action of the current, all contraction in the limb traversed by the direct current will be seen to disappear, whilst the contraction becomes stronger in the limb traversed by the inverse current. The contraction, which, in the commencement, manifested itself at the closing in the limb in which the direct current circulated, is no longer induced even at the closing, and in the limb traversed by the inverse current, the contraction which occurred only at the opening of the current, is induced now, not only at the opening, but even at the closing.

Moreover, by acting directly upon the nerves with an electric current, or with a mechanical or chemical irritant, it is always demonstrated that the nerve which has been traversed by the direct current has lost its excitability, whilst the excitability of the nerve of the opposite leg, which was under the influence of an inverse current, has not only been preserved, but has been perceptibly augmented.

Hence, *the excitability of nerves is diminished by a direct or descending, and increased by an inverse or ascending current.* As a consequence of this law, we have, moreover, the following proposition, which is further confirmed by experiment: *A nerve fatigued by the descending, regains its excitability by the ascending current, and a nerve whose excitability has been augmented by the ascending, loses it by the descending current*, (Volta, Lehot, Marianini.) In the case of very vigorous frogs, which have been subjected during a certain length of time to the passage of the current, it often happens that the contractions awakened in the inverse limb by the opening of the circuit, is not an instantaneous phenomenon, but a tetanic condition which persists during several seconds, (Ritter.) It is sufficient in this case, in order to arrest the tetanic contractions, to re-establish the primitive current.

The cause of the augmentation of the excitability of nerves, under the influence of the ascending current, depends at the same time upon the nerve and the cord.

In the frog prepared after the manner of Galvani, the nerves of the lower limbs remain attached to the cord, and the excitability of the nerve on the side of the limb traversed by the ascending current may then depend upon the influence of the spinal center. This influence is real, as we have been able to demonstrate. By destroying the cord completely by means of a stylet, and by sending through it, under these conditions, a current from one limb to the other, there was still observed a greater excitability in the nerve traversed by the inverse than in that traversed by the direct current, but its excitability is much less augmented than in the case in which the cord is intact.

The influence of the cord acts, then, to increase the excitability of peripheral nerves traversed by an inverse current: but this augmentation is equally due to the direct action of the electrical currents upon the nerves. Matteucci has made the following experiments: In a frog which exhibited clearly the tetanic condition in the inverse limb at the opening of the circuit, after having left the circuit closed during a certain time, he cut the nerve, either near the spinal cord, or, on the contrary, at the point where it enters into the muscle of the thigh. In the first case, the interruption of the circuit thus produced excited, as usual, tetanic contraction, whilst nothing happened when the nerve had been entirely removed. This experiment proves that the presence of the nerve is necessary to produce this phenomenon, and, moreover, that the direct modification sustained by the nerve is the cause of this tetanic state.

Let us add this fact, which we have determined, and which is important in explanation of the phenomenon, as we shall see further, that when the nerve is cut between the two poles, no contraction, or at least no tetanic contraction, supervenes.

